# Single‐use versus multiple‐use endotracheal suction catheters flushed with chlorhexidine in mechanically ventilated ICU patients: A study protocol of a feasibility randomized controlled trial with an embedded qualitative study

**DOI:** 10.1111/nicc.13227

**Published:** 2025-01-02

**Authors:** Mohamed H. Eid, Kevin Hambridge, Pat Schofield, Jos M. Latour

**Affiliations:** ^1^ Faculty of Health, School of Nursing and Midwifery University of Plymouth Plymouth UK; ^2^ Critical Care and Emergency Nursing Department, Faculty of Nursing Mansoura University Mansoura Egypt; ^3^ Department of Nursing, Zhongshan Hospital Fudan University Shanghai China; ^4^ The Curtin School of Nursing Curtin University Perth Australia

**Keywords:** airway management, chlorhexidine, endotracheal suctioning, ventilator‐associated pneumonia, green ICU

## Abstract

**Background:**

Endotracheal suction catheters are often used multiple times during endotracheal suctioning procedures in resource‐limited intensive care units (ICU). The impact of this practice on mechanically ventilated patients' outcomes remains unclear.

**Aim:**

The aim of this feasibility randomized controlled trial (fRCT) is to assess the feasibility and acceptability of single‐use versus multiple‐use endotracheal suction catheters flushed with chlorhexidine in mechanically ventilated ICU patients.

**Study Design:**

This study is a three‐armed fRCT with an embedded qualitative study.

**Results:**

The trial involves three groups. One group includes endotracheal suctioning using a single‐use catheter; the second group includes a multiple‐use endotracheal suction catheter flushed with chlorhexidine and the control group includes a multiple‐use endotracheal suction catheter flushed with normal saline. Sixty adult ICU patients (20 in each group) will be recruited, along with 12–16 ICU nurses delivering the interventions, and 12–16 patients' next‐of‐kin for semi‐structured interviews. The study protocol has been approved by two ethics committees. Study recruitment will be conducted over an 8‐month period with an expected start date of 12 April 2024.

**Conclusion:**

The feasibility outcome measures will be recruitment, retention, and follow‐up measures as well as the identification of clinical outcomes such as Ventilator‐Associated Pneumonia (VAP) using the modified clinical pulmonary infection score, and ICU length‐of‐stay.

**Relevance to Clinical Practice:**

This study will help ICU nurses to understand how different methods of endotracheal suctioning affects patients in ICUs with limited resources. The findings could influence clinical practice and improve patient outcomes.

**What is known about this topic**

- Endotracheal suction catheters are commonly used multiple times in resource‐limited ICU settings.
- The practice of reusing endotracheal suction catheters and its impact on patient outcomes is not well understood.

**What this paper adds**

- This feasibility randomized controlled trial will evaluate the feasibility of single‐use versus multiple‐use endotracheal suction catheters flushed with chlorhexidine in an ICU setting.
- The trial will provide preliminary data on clinical outcomes, including VAP incidence and ICU length‐of‐stay, to inform future large‐scale trials and potential changes in clinical practice to improve patient care in resource‐limited ICU settings.

## 1 INTRODUCTION

Ventilator‐associated pneumonia (VAP) is a significant healthcare‐associated infection occurring in patients on mechanical ventilation for over 48 h.^1^, ^2^ VAP is a problematic, life‐threatening, intensive care unit (ICU) issue worldwide. It can prolong ICU length‐of‐stay, duration of mechanical ventilation, and can increase patient morbidity, and healthcare costs.^3^ A narrative review reported that 5%–40% of patients who are on a mechanical ventilator for more than 2 days develop VAP.^4^ The estimated attributable mortality of VAP is approximately 10%, with higher mortality rates in surgical ICU patients.^4^ VAP is categorized into early onset (within 4 days of mechanical ventilation) and late onset (Day five or more).^1^, ^5^ *Staphylococcus aureus*, *Klebsiella* spp., *Pseudomonas aeruginosa*, *Acinetobacter* spp. are the most common pathogens, causing around 80% of hospital acquired pneumonia.^6^

Diagnosing VAP is challenging because of its non‐specific symptoms, often overlapping with other respiratory conditions. Effective prevention includes reducing the risk of aspiration and colonisation of pathogens in the lower respiratory tract.^7^ Therefore, endotracheal suctioning to remove respiratory secretions is essential in the respiratory management in mechanically ventilated patients.^8^, ^9^ This procedure not only removes pulmonary secretions but also enhances oxygenation and ventilation, and prevents complications like pneumonia, atelectasis, and tube blockage.^10^ Suctioning should be performed as needed rather than on a fixed schedule, guided by indicators such as visible secretions, coughing, and audible crackles over the trachea.^11^ It is a sterile procedure requiring adherence to specific principles to minimize lung contamination, as outlined in recent guidelines from the American Association of Respiratory Care in 2022.^12^ Nurses are crucial in applying sterile principles before, during, and after suctioning to ensure patient safety and effective respiratory care.^13^

A suction catheter is essential for removing tracheal secretions and can be part of an open or closed endotracheal suctioning system. The open system involves briefly disconnecting the ventilation circuit to insert a single‐use catheter,^14^ whereas the closed system keeps the catheter sterile within an enclosed sheath connected to the endotracheal tube, whilst avoiding disconnection.^11^ Recent studies indicate no significant difference in VAP incidence or mortality rates between open and closed systems.^14^, ^15^, ^16^ Additionally, changing closed suction catheters every three versus 7 days has not affected VAP rates in one randomized trial.^17^ Evidence suggests that reusing suction catheters in both systems do not increase VAP incidence, and there is no evidence/guideline for using a new catheter for each suction procedure.^18^ Therefore, the evidence for reusing suctioning catheters multiple times remains unclear.

Chlorhexidine is a widely used, well‐known, and low‐cost disinfectant that kills most pathogenic organisms and subsequently reduces the spread of hospital‐acquired infections in ICUs.^19^ It is recommended by the American Thoracic Society (ATS), Centres for Disease Control and Prevention (CDC), and Infectious Diseases Society of America (IDSA). Chlorhexidine has a slow‐release antibacterial activity that extends up to 48 h after its initial application, which explains the reason why organisms that encounter chlorhexidine after its application may not be able to grow.^20^

In resource‐limited countries, endotracheal suctioning is often performed using endotracheal catheters multiple‐times during a nursing shift. The World Health Organization (WHO), describes resource‐limited settings as locations where ‘health systems are weak due to a lack of resources, including skilled health workers, finances, and infrastructure’.^21^ Resource limitations are common in low‐ and middle‐income countries, but they can also be found in rural or underserved areas within high‐income countries.^21^ The incidence of VAP remains high in resource‐limited ICU settings.^22^ It is unclear whether using single‐use catheters or multiple‐use catheters flushed with chlorhexidine reduces VAP incidence. Therefore, we designed a feasibility randomized controlled trial (fRCT), with an embedded qualitative study to explore this issue.

### 1.1 Aim and objectives

The overall aim of the fRCT is to determine the feasibility and effect of single‐use versus multiple‐use endotracheal suction catheters flushed with chlorhexidine on VAP incidence in mechanically ventilated patients, and to determine methods for the design of a definitive multicentre RCT. The fRCT's specific objectives are presented in Box 1.

BOX 1 **Objectives of the fRCT.**

The objectives a–d will be met within the fRCT study:

- Assess the feasibility and acceptability of the trial procedures by comparing the effect of single‐use endotracheal suction catheters versus multiple‐use endotracheal suction catheters flushed with chlorhexidine in mechanically ventilated patients.
- Select primary and secondary outcome measures which are most suitable for informing the calculation of sample size for the definitive RCT.
- Identify confounding factors which might affect the future trial.
- Assess the patients' recruitment technique, follow‐up rate, and any unexpected side effects of the intervention.

The objectives e‐i will be met within the embedded qualitative study:

e To evaluate the willingness of clinicians to recruit, identify, and randomize the eligible patients for the trial.
f To explore the acceptability of the interventions delivered by the ICU nurses in the study setting.
g To explore the experiences of ICU nurses using chlorhexidine for flushing the suctioning circuit after the endotracheal suction procedure.
h To explore the experiences of ICU nurses using a single‐use endotracheal suction catheter.
i To explore the experiences of patients' families (next‐of‐kin) of the recruitment process, the process of deferred consent, and being involved in the trial.

## 2 METHODS

The study design is a three‐armed fRCT with an embedded qualitative study aiming to determine the feasibility of single‐use versus multiple‐use endotracheal suction catheters flushed with chlorhexidine in mechanically ventilated patients, and to determine methods for the design of a definitive RCT. The protocol of this fRCT has been registered prospectively at ClinicalTrials.gov NCT06207513. This study protocol is reported in accordance with the SPIRIT guidelines (Supplementary Material I). Upon completion, the study will be reported using the CONSORT 2010 statement: extension for randomized pilot and feasibility trials.^23^

### 2.1 Setting

This study will be conducted in three ICUs within a single university hospital in Egypt, comprising surgical, neurological, and trauma ICUs. Each ICU has 10 beds and provides care to mechanically ventilated patients. These units are well equipped with advanced technologies and resources necessary for delivering high‐quality ICU care. The total admissions for the three ICUs are approximately 650 patients annually. The nurse–patient ratio in these units is one nurse caring for two patients.

### 2.2 Participants

Adults aged 18 years and older, newly admitted to the ICU, intubated with an endotracheal tube, and undergoing mechanical ventilation for at least 48 h will be included in the study. Exclusion criteria are: (1) Patients who have received standard ICU care with multiple endotracheal suction procedures; (2) patients with contraindications to suctioning such as elevated intracranial pressure, severe haemoptysis, and cerebrospinal fluid leaks; (3) previously intubated patients during the current hospital stay; (4) patients expected to require mechanical ventilation for less than 72 h; (5) patients diagnosed with pneumonia upon ICU admission or with a Modified Clinical Pulmonary Infection Score (MCPIS) of 5 or higher; (6) patients with conditions such as atelectasis, acute respiratory distress syndrome (ARDS), and pulmonary oedema; (7) patients known to be allergic to chlorhexidine; and (8) patients whose family members (next‐of‐kin) have not given deferred consent within 48 h of ICU admission.

### 2.3 Sample size

As the study design is a fRCT, no formal power calculation will be undertaken.^24^ The feasibility study's recommended number of participants ranges between 20 and 70.^24^ Therefore, this fRCT (quantitative part) will be conducted among mechanically ventilated patients using a convenience sample of 60 adult ICU patients: 20 patients in each study arm.

### 2.4 Trial design

**FIGURE 1:**
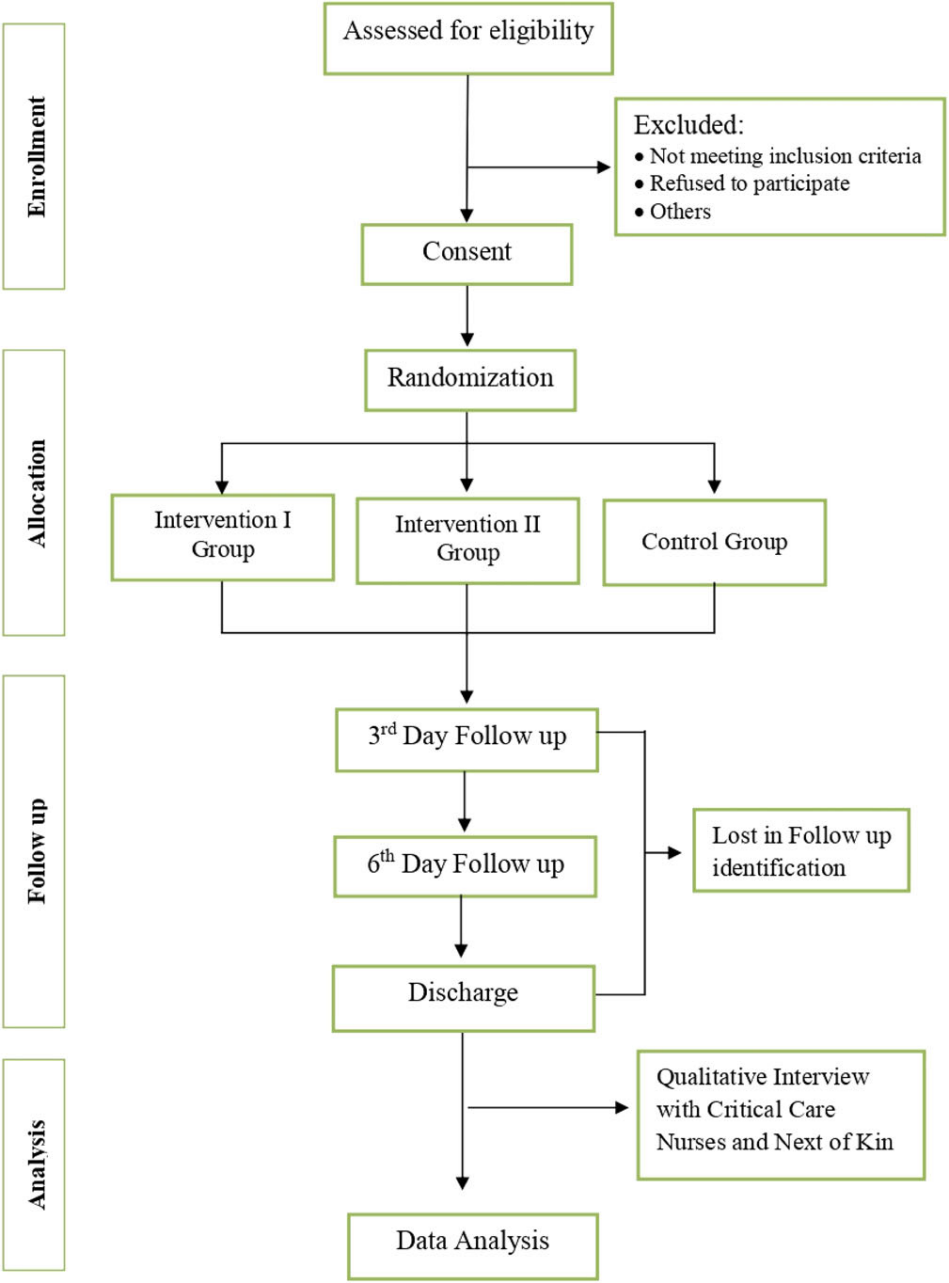
The fRCT study design.

The study procedure is summarized in Figure 1. Study participants will be randomly assigned upon ICU admission to one of three groups:

Group I: Single‐used endotracheal suction catheter (Intervention I group).

Group II: Multiple‐used endotracheal suction catheter flushed with chlorhexidine after every endotracheal suctioning procedure (Intervention II group).

Group III: Standard care, multiple‐used endotracheal suction catheters flushed with normal saline after every endotracheal suctioning procedure (Control group).

The trial will use a sealed envelope system for randomisation, which will be evaluated for the future RCT. At this stage, no stratification based on variables such as age or disease type (e.g., high vs. low mucus production) has been incorporated. However, we recognize the potential value of stratified randomisation and will consider evaluating this approach in future iterations, particularly for the subsequent randomized controlled trial, where stratification may enhance the precision of the findings.

Blinding is not feasible due to the differences in chlorhexidine and saline bottle shapes and colours. The clinical lead nurse will manage patient screening, consent, and recruitment, whilst the principal investigator (PI) collects outcome measures. Critical care nurses trained by the PI will deliver the interventions.

### 2.5 Recruitment

**FIGURE 2:**
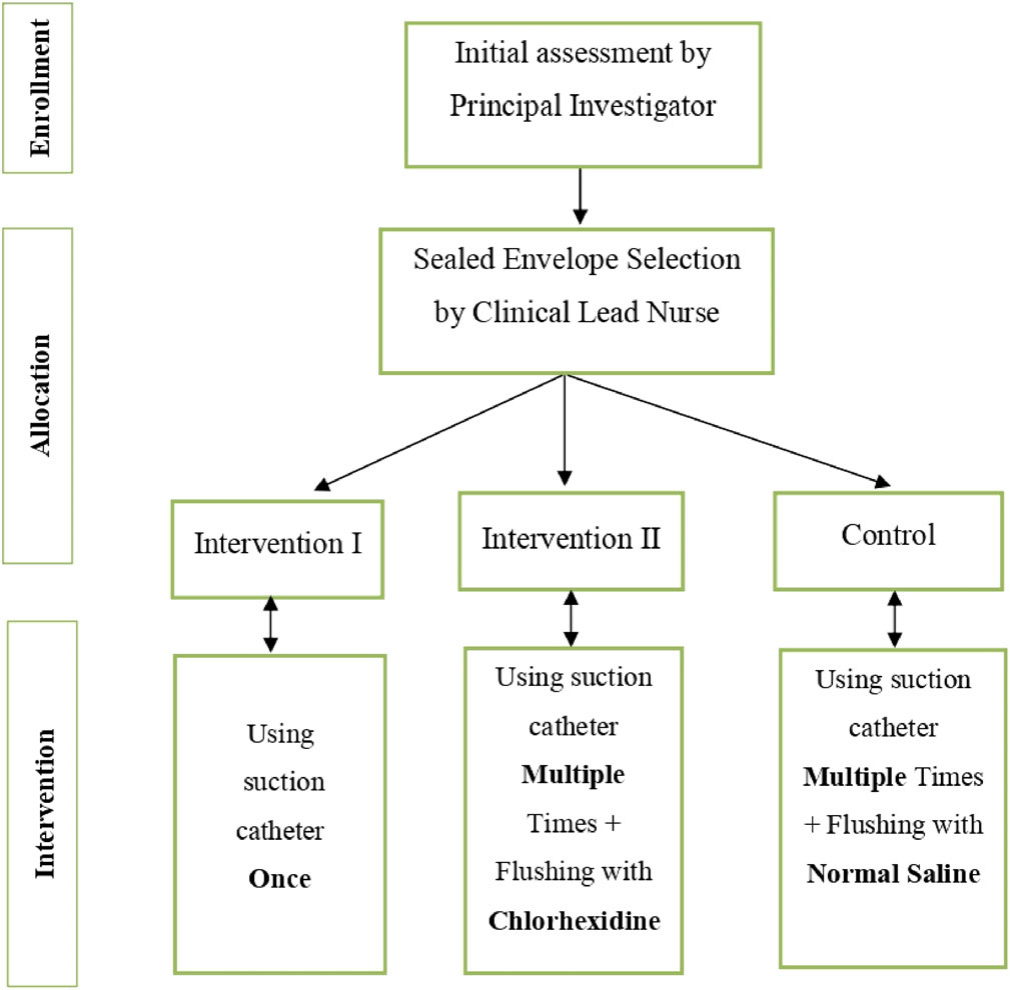
The study recruitment process.

The enrolment and allocation process are detailed in Figure 2. The clinical lead nurse or PI will recruit and assess newly admitted mechanically ventilated ICU patients on their first day using the MCPIS to confirm eligibility. Allocation will occur before the first suctioning procedure to avoid contamination.

### 2.6 Nurse training

The PI will offer training to critical care nurses at the study sites prior to implementing the intervention. Once the PI confirms the nurses' competency, they can commence the intervention delivery. Additional information regarding this training is available in Supplementary Material II.

### 2.7 Interventions, standard care and procedures

The PI will be responsible for collecting data which will be done through three phases including assessment, implementation, and evaluation.

### 2.8 Assessment phase

During this phase, the PI will perform a first‐day initial assessment for all ventilated patients using MCPIS to confirm the absence of pneumonia and preselected exclusion criteria using tool II ‘VAP diagnostic criteria sheet’. Patients who have pneumonia on admission will be excluded from the study, and recruitment will include patients who fulfil the study inclusion criteria.

### 2.9 Implementation phase

Following the assessment phase, the PI will inform the Critical Care Nurses to initiate the interventions of this study after reviewing current practices, the literature review, and clinical practices related to endotracheal suctioning.^11^, ^14^, ^15^, ^16^, ^17^

The researchers will refrain from creating a suctioning performance checklist based on established guidelines or asking the staff nurses to modify their current suctioning practices in either the study or control groups. Suctioning will be performed based on indications such as the presence of secretions in the patient's chest and auscultation findings of crackles or wheezing.

#### 2.9.1 Intervention I group

The PI will ask the staff nurses to perform suctioning using a single‐use open endotracheal suction catheter for one‐time suctioning attempts, discarding each catheter after use. If additional suctioning is required, a new catheter will be used and then discarded. Patients in this group will be identified by a red label on their records to ensure consistent intervention assignment.

#### 2.9.2 Intervention II group

The PI will ask the staff nurses to use open endotracheal suction catheters multiple times during 12‐h shifts, with one catheter for day and another for night use. Flushing of the suctioning circuit will be performed with 40 mL of chlorhexidine gluconate 0.2%. The nurses will perform a brief 5‐s ‘dry suctioning’ before reuse to ensure no chlorhexidine remains in the catheter, preventing inadvertent lung instillation. Patients in this group will be identified with a yellow label on their records to ensure consistent intervention assignment.

#### 2.9.3 Control group

The PI will ask the staff nurses to perform their standard suctioning procedure using an open endotracheal suction catheter that is used multiple times during a 12‐h nursing shift. One catheter will be used for a day shift and another catheter for a night shift. Flushing of the suctioning circuit will be performed using routine normal saline. To maintain consistency in care, these patients will have a green label on their records indicating their assigned intervention group.

### 2.10 Evaluation phase

Feasibility and acceptability of conducting the intervention among the nursing staff will be evaluated. Additionally, MCPIS will be calculated for each patient in each study group. A comparison will be done between the three groups to evaluate the effect of the interventions on the occurrence of VAP on Day three (early VAP) and Day six (late VAP) using tool ΙΙ ‘VAP Diagnostic Criteria Sheet’. Another comparison will be conducted within the study participants of the three groups to investigate other implications on mechanically ventilated patients' outcomes, including length of ICU stay and mortality.

### 2.11 Data collection tools

Data collection in this study will involve three distinct tools (Supplementary Material III), incorporating both quantitative and qualitative methods. Quantitative data will primarily be obtained from participant patients using two specific tools, as summarized in Box 2.

BOX 2 **Summary of quantitative data collection tools.**

Tool Ι: Mechanically Ventilated Patients Assessment Tool

This tool, developed by the Principal Investigator after thorough literature review, is structured into three parts:

Part Ι: Patient's Sociodemographic and Health Relevant Data

This section will capture sociodemographic details such as gender, age, occupation, and smoking status. It will also include critical health information such as ICU admission date, reason for admission, medical history, diagnosis, length of ICU stays, and Modified Glasgow Coma Scale (MGCS) score.

Part ΙΙ: Ventilator Modalities Data

Here, data will be recorded on the initiation date of mechanical ventilation, use of artificial airway, size of the endotracheal tube, and specifics regarding mechanical ventilation mode and duration.

Part ΙΙΙ: Endotracheal Suctioning Data

This part will document details pertinent to the endotracheal suctioning procedure, including suction catheter size, catheter connector type, total procedure time, whether single‐use or multiple‐use suction catheters used, and the type of flushing material used (normal saline or chlorhexidine).

Tool ΙΙ: VAP Diagnostic Criteria Sheet

This tool, adopted from Singh et al.,^25^ uses the MCPIS for VAP diagnosis. The original Clinical Pulmonary Infection Score (CPIS) by Pugin et al.^26^ included six clinical assessments: body temperature, tracheal secretions, WBC count, oxygenation, chest radiography, and sputum culture. Because of delays in culture results, Singh et al. proposed the MCPIS including the first five variables for initial diagnosis. The MCPIS scores range from 0 to 10, with scores above five suggesting pneumonia. Borderline scores (5) and haemodynamically stable patients are reassessed on Day 8; unstable patients undergo sputum culture on Day 6. Negative cultures contribute zero points, whilst positive cultures add two points, indicating VAP (MCPIS = 7). Chest x‐rays will be validated with the PI and the clinical lead nurse. In case of confusion the ICU consultant will be involved.

### 2.12 Outcomes measures

The outcomes of the study focus on the feasibility of the intervention, the feasibility of conducting the trial, and patient outcomes (Box 3).

BOX 3 **The outcomes of the study.**

Feasibility of the intervention

- Evaluating the feasibility of using chlorhexidine as a flushing solution of endotracheal suction circuit in the ICU.
- Exploring the feasibility of using the suction catheter a single time for suctioning procedure.
- Exploring any potential adverse effects of the interventions.

Feasibility of conducting the trial

d Evaluating flexibility with resources utilisation (suction catheters, saline, and chlorhexidine), for the conduction trail, and the number of eligible, recruited, and withdrawn patients.
e Identifying confounding factors which might affect the proposed trial (i.e., age, severity of disease, and underlying diseases).

Patients' outcomes

f Ventilator‐associated pneumonia (early VAP will be measured at Day three of mechanical ventilation, and late VAP will be measured at Day six).
g Length of ICU stays and mortality.

Assessing different potential primary and secondary outcomes of the future trial

h Investigating the impact of the proposed interventions on health economics.

### 2.13 Progression criteria

Progression to a full RCT will be considered if the following minimum success criteria are met, or if there is reason to believe that appropriate improvements can be made to address any concerns in the full trial:

- The number of recruited patients, nurses, and next‐of‐kin
- Retention of at least 75% of participants over the trial duration.
- The willingness and acceptability of the proposed intervention in study settings
- Completion of primary and secondary outcome measures by 80% of participants

### 2.14 Statistical analysis

As a feasibility study it would be inappropriate to test treatment/intervention effects. Therefore, the statistical analysis will be descriptive in design. The aim of the analysis is to assess the feasibility of the intervention, the feasibility of a full definitive trial, and to summarize potential primary and secondary outcome measures.

To identify the patients' outcomes, quantitative data collected will be coded, processed, and analysed statistically using IBM SPSS Statistics for Windows, version 24 (IBM Corp., Armonk, N.Y., USA). Results will be presented as frequencies and percentages for categorical variables, and as mean with standard deviation for continuous variables.

## 3 EMBEDDED QUALITATIVE STUDY

This component of the trial explores the experiences of the ICU nurses who will deliver the intervention along with patients' families (next‐of‐kin). Semi‐structured interviews will be conducted with ICU nurses to investigate the delivery and acceptability of the interventions and the ICU nurses' experience of using chlorhexidine for flushing the suctioning circuit and the use of single‐use endotracheal suctioning catheters. The data will be collected from ICU nurses who delivered the study interventions and have been involved in the care of the study participants in one of the three study settings. Additionally, a sample of next‐of‐kin will be interviewed for their experience of allowing their patient to participate in the study.

### 3.1 Sampling

A convenience sample of 12–16 ICU nurses and 12–16 next‐of‐kin will be invited to the semi‐structured interviews.

### 3.2 Qualitative data collection

Tool III, including an interview guide, will be used to collect qualitative data consisting of two parts: part I, questions for ICU nurses and part II questions for next‐of‐kin regarding their experience in participating in the trial. (Supplementary Material II).

### 3.3 Qualitative data analysis

Thematic analysis will be used to analyse qualitative data by identifying themes and subthemes as proposed by Braun and Clarke.^27^ Interview transcripts will be managed using NVivo software. To reduce bias, two researchers will independently code the transcripts, deriving codes from text segments that may be revised during analysis. Researchers will then compare and discuss their coding to reach consensus. Findings will follow the COREQ checklist for qualitative research.^28^

### 3.4 Data management plan

The data management plan for our trial can be found in Supplementary Material IV.

### 3.5 Compliance

Reasons for non‐adherence to the study protocol will be investigated and addressed. Nurse interviews conducted as part of the embedded qualitative component will assist in identifying potential reasons for non‐compliance. Any logistical issues within hospital policy that impact the delivery of the interventions and standard care will also be documented.

### 3.6 Unblinding

Unfortunately, blinding will not be feasible within the fRCT for two reasons. Firstly, the shape of the chlorhexidine bottles is very different to the shape of normal saline bottles, and the colour of the solution is slightly different. Secondly, one arm of the study includes using the suction catheter once which will be different from the other two groups that will use the catheter multiple times. Therefore, we will ensure the randomisation of study participants for accuracy and fidelity as blinding is not feasible.

### 3.7 Consent

Ethical approval has been obtained from the Faculty Research Ethics and integrity Committee (FREIC), University of Plymouth, United Kingdom (Reference: 4333) and the Research Ethics Committee at Faculty of Nursing, Mansoura University, Egypt (Reference: 411/2023). An official permission from the hospital's administrative authority to conduct the study has been obtained (27 November 2023). Families (next‐of‐kin) will be provided with written information regarding the aim of the study, the nature of the suctioning procedure, expected benefits, and the risks. The voluntary nature of participation and confidentiality will be maintained.

### 3.8 Deferred consent

Because of the nature of mechanically ventilated patients, obtaining written consent is often not possible, such as when patients are sedated. To ensure unbiased adherence to the intervention, such as using single‐use catheters or chlorhexidine flushing, allocation to study groups will be overseen by the clinical lead nurse upon ICU admission and before suctioning. The next‐of‐kin will receive an information sheet (Supplementary Material III), within 24 h to decide on deferred written consent. If consent is declined, patients will receive standard ICU care, maintaining ethical standards.

### 3.9 Withdrawal criteria

Each participant's next‐of‐kin retains the complete right to voluntarily withdraw their patients from the study at any point, without any obligation. Instances where this might happen include adverse events experienced by patients. In such cases, the patient will be withdrawn from the study and will not be considered for recruitment into any study groups in the future.

### 3.10 End of trial

The fRCT will end on the date of the last follow‐up of the final participant. Premature termination of the trial may occur under the following circumstances:

- An unacceptable frequency of adverse events.
- A fully powered RCT evaluating a similar intervention with comparable participants. As of 1 June 2024, no similar trials are registered on clinicaltrials.gov.
- Administrative decision made by the hospital due to a fatal case report or unexpected outcomes.

### 3.11 Limitations

This study will be conducted within a single hospital and will utilize a relatively small sample size. Consequently, the findings may have limited generalisability. The primary objective is to assess the feasibility of conducting the study within this specific setting. Future research is needed to include a larger, more diverse sample, and considering a multicentre approach to enhance the robustness and applicability of the results. Another limitation is that the incidence of VAP will be assessed using the MCPIS, however, the alveolar bronchoscopy is the accurate test for detecting VAP (diagnosis with BAL >104 CFU/mL).

### 3.12 Conclusion

In conclusion, the aim of this fRCT is to assess the practicality and impact of using single‐use versus multiple‐use endotracheal suction catheters flushed with chlorhexidine on the incidence of VAP in mechanically ventilated patients. The study is expected to provide crucial insights into the feasibility of a larger definitive trial and offers valuable data on the effectiveness and acceptability of the interventions in resource‐limited ICU settings, potentially informing future clinical practices.

## AUTHOR CONTRIBUTIONS

MHE contributed to all aspects of the study protocol. JML contributed to the design of the study including the methodology. KH, PS contributed to background and structuring of the quantitative and qualitative components. All authors contributed to the revisions of the manuscript, and all authors approved the final version.

## FUNDING INFORMATION

The researcher (Mohamed H. Eid) is funded by a PhD scholarship (MM71/22) from the Ministry of Higher Education of the Arab Republic of Egypt.

## CONFLICT OF INTEREST STATEMENT

The authors declare no potential conflicts of interest concerning the research, publication, and/or authorship of this article.

## ETHICS STATEMENT

The study is reviewed and approved by the following ethical committees: Faculty of Health Research Ethics and Integrity Committee at University of Plymouth, United Kingdom (Reference: 4333, 14 December 2023) and the Research Ethics Committee at Faculty of Nursing Mansoura University, Egypt (Reference: 411/2023, 27 November 2023).

## PATIENT CONSENT STATEMENT

The research ethics committees mentioned in the paragraph above have reviewed and approved the informed consent forms and participants information sheets for this study.

## Supporting information


**Data S1:** Supporting Information.

**Data S2:** Supporting Information.

**Data S3:** Supporting Information.

**Data S4:** Supporting Information.

## Data Availability

The data that support the findings of this study are available from the corresponding author upon reasonable request.
