# Supplementary material for: Single‐use versus multiple‐use endotracheal suction catheters flushed with chlorhexidine in mechanically ventilated ICU patients: A study protocol of a feasibility randomized controlled trial with an embedded qualitative study
Source: Nurs Crit Care. 2025 Jan 2;30(4):e13227. doi: 10.1111/nicc.13227 (PMC12234884; doi:10.1111/nicc.13227)
Supplement: Supplementary file 2 — Data S2: Supporting Information. [file NICC-30-0-s001.docx]

**Single-used versus multiple-used endotracheal suction catheters flushed with chlorhexidine in mechanically ventilated ICU patients: A study protocol of a feasibility randomised controlled trial with an embedded qualitative study**

## Supplementary Material I: Nurses Training Package

- Training session will last for 60 minutes; first 30 minutes will be in the staff room and includes theoretical explanation of study proposed interventions for nurses.
- Followed by 30 minutes hands on simulation training and real-world training in intensive care units.
- Clinical nurse lead and PI will observe nurses’ interventions.
- Once the PI and clinical nurse lead agreed that the nurse is competent in performing the proposed interventions, the nurse will be enrolled in study participating staff.
- Training will be carried out one week prior the start of the study to train most ICU nurses.
- We will record the name of trained nurses.
- The training session will use the standard/routine endotracheal suctioning protocol of the ICU.
- Below is the summary for the proposed intervention for each study group:

**Intervention I Group (using suction catheter once)**

1. Perform endotracheal suctioning for the patient following hospital protocol.
2. Discard the catheter once you removed it out of patients’ endotracheal tube.
3. Attach a new catheter to the suction circuit.
4. Insert the new catheter into patient’s endotracheal tube and start suctioning again.
5. Never re-use the suction catheter once you take it out of patient.

**Intervention II (using suction catheters multiple times + flushing with chlorhexidine)**

1. Pour 40 ml of chlorhexidine bottle into a sterile container.
2. Perform endotracheal suctioning for the patient, you will use the suction catheter multiple times.
3. Once you finished suctioning, use the 40mls of chlorhexidine to flush the suction circuit.
4. In case secretions remains in patient’s chest, suction again and use another 40 mls of chlorhexidine for flushing.
5. If you find secretions still stuck in patient’s suctioning circuit, use another 40 mls of chlorhexidine for flushing.

**Control group (standard care; using suction catheters multiple times + flushing with Saline)**

1. Perform endotracheal suctioning for the patient according to hospital policy, you will use the suction catheter multiple times.
2. Once you finished suctioning, use the normal saline to flush the suction circuit.
