# Supplementary material for: Single‐use versus multiple‐use endotracheal suction catheters flushed with chlorhexidine in mechanically ventilated ICU patients: A study protocol of a feasibility randomized controlled trial with an embedded qualitative study
Source: Nurs Crit Care. 2025 Jan 2;30(4):e13227. doi: 10.1111/nicc.13227 (PMC12234884; doi:10.1111/nicc.13227)
Supplement: Supplementary file 3 — Data S3: Supporting Information. [file NICC-30-0-s002.docx]

**Single-used versus multiple-used endotracheal suction catheters flushed with chlorhexidine in mechanically ventilated ICU patients: A study protocol of a feasibility randomised controlled trial with an embedded qualitative study**

## Supplementary Material II: Data Collection Tools

- **Patient's Name:**

**Patient's Code:**

- **Admission Number:**
- **Study Group:**

| **Intervention I** |
| --- |
| **Intervention II** |
| **Control** |

**Tool I: Mechanically Ventilated Patient Assessment Tool**

**Part 1: Patient's Socio-demographic and Health Relevant Data**

1. **Patient's Characteristics:**

| 1. **Age:**   18-20  31-40  51-60 | | |  | 21- 30  41-50  Above 60 |  |
| --- | --- | --- | --- | --- | --- |
| 1. **Gender** | | |  |  |  |
| Male | | |  |  |  |
| Female | | |  |  |  |
| Other | | | Specify… | |  |
| 1. **Occupation:** | | |  |  |  |
| Employee | | |  | Farmer/Worker |  |
| Housewife | | |  | Retired |  |
| Other (specify): | | |  |  |  |
| 1. **Smoking Habits:** | | |  |  |  |
| Current Smoker |  | Past Smoker |  | Non - Smoker |  |

1. **Health Relevant Data:**

| 1. **Date of admission to ICU: / /** | |
| --- | --- |
| 1. **Reason for admission:** | |
| Respiratory Failure | Multiple Injury |
| Cardiac Disease | Renal Disease |
| Neurological Disease | Others Specify……………… |
| 1. **Medical diagnosis:** |  |
| 1. **Past medical history:** |  |
| Diabetes Mellitus | Hypertension |
| Ischemic heart disease | Renal failure |
| Hepatic impairment | Others specify……………… |

1. **Duration of ICU Stay**

| 1-2 days | 3-4 days |
| --- | --- |
| 5-6 days | ≥7 days |

1. **Level of Consciousness Based on Modified Glasgow Coma Scale (MGCS):**
2. **On admission:**
3. **Daily assessment:**

| Day/shift | 1^st^ day | 2^nd^ day | 3^rd^ day | 4^th^ day | 5^th^ day | 6^th^ day |
| --- | --- | --- | --- | --- | --- | --- |
| MGCS |  |  |  |  |  |  |

**Part II: Ventilator Modalities Data**

1. **Mechanical Ventilation Initiation Date:**
2. **Artificial airway**

| Endotracheal Tube | Tracheostomy |
| --- | --- |
| Laryngeal Mask | Others |

1. **Intubation Process**

| Urgent | Elective |
| --- | --- |

1. **Size of Endotracheal Tube**

| 5-5.5 | 6-6.5 |
| --- | --- |
| 7-7.5 | 8-8.5 |

1. **Mode of Ventilation**

| Controlled |
| --- |
| Assisted |
| Spontaneous |

1. **Duration of Mechanical Ventilation**

| 1-2 days | 3-4 days |
| --- | --- |
| 5-6 days | ≥7 days |

**Part III: Endotracheal Suctioning Data**

| 1. **Size of Suction Catheter** |  |
| --- | --- |
| ≤10 Fr | 12 Fr |
| 14 Fr | 16 Fr |
| 18 Fr | 20 Fr |
| 1. **Type of Suction Catheter Connector** | |
| Standard Connector |  |
| Thumb Control Connector |  |
| Fingertip Control Connector |  |
| 1. **Duration of Total Suction Time** |  |
| ˂ 30 Seconds |  |
| 30 Seconds -1 Minute |  |
| ˃ 1 Minute |  |
| 1. **Frequency of Using Suction Catheter** |  |
| Single-used | Multiple Used |
| 1. **Flushing Solution Used** |  |
| Normal Saline | Chlorhexidine |

**Tool ΙΙ: VAP Diagnostic Criteria Sheet**

**The Modified Clinical Pulmonary Infection Score**

**(Modified CPIS)**

| **CPIS Elements** | **Range** | **Score** | **Day 1** | **Day 3** | **Day 6** |
| --- | --- | --- | --- | --- | --- |
| **Temperature** | ≥ 36.5 and ≤ 38.4 | 0 |  |  |  |
|  | ≥ 38.5 and ≤ 38.9 | 1 |  |  |  |
|  | ≥ 39 and ≤ 36 | 2 |  |  |  |
| **Blood Leukocytes Count, per mm^3^** | 4,000 – 11,000 | 0 |  |  |  |
|  | ˂4,000 or ˃11,000 | 1 |  |  |  |
|  | ˂4,000 – ˃11,000 + band forms ≥ 500 | 2 |  |  |  |
| **Tracheal Secretions** | Rare | 0 |  |  |  |
|  | Abundant | 1 |  |  |  |
|  | Abundant + Purulent | 2 |  |  |  |
| **Oxygenation PaO_2_/FiO_2_** | ˃ 240 or ARDS | 0 |  |  |  |
|  | ≤ 240 and no evidence of ARDS | 2 |  |  |  |
| **Chest X-ray Infiltrates** | No infiltrates | 0 |  |  |  |
|  | Diffused | 1 |  |  |  |
|  | localized | 2 |  |  |  |
| **Total Score** | **˃ 5 = VAP** |  |  |  |  |

**Tool ΙIΙ, Interview Guide, [Part I]: Nurses Semi-Structured Interview Questions Tool**

| **Questions** | **Prompts** | **2^nd^ Prompts** |
| --- | --- | --- |
| Think back to your standard routine of suctioning using suction catheters multiple times and use normal saline for flushing the suction circuit of patient’s secretions. Did you feel that the standard care has an impact on mechanically ventilated patients’ outcomes? | If Yes- can you describe if it has a good or a bad effect on your patient? (In what ways did the patients affected)  If no- why not? What would you have liked about the routine care? | *Prompt-including nurses’ beliefs about routine endotracheal suctioning.* |
| Did you have negative, challenging, or troubling thoughts or feelings surrounding the need for changing the routine suctioning care and try new suctioning modalities? | What did you like or dislike about the information regarding trying a new suctioning technique? |  |
| Primary outcomes (3^rd^ day follow up)  Think back to the day three of applying the intervention. Was your patient’s status improved or deteriorated in each study groups? | Were the outcomes good or bad? And was it related to a specific study intervention? | If it was bad, was it related to a specific group or all groups? |
| Primary outcomes (6^th^ day follow up)  Think back to the day six of applying the intervention. Was your patient’s status improved or deteriorated in each study groups? | Were the outcomes good or bad? And was it related to a specific study intervention? | If it was bad, was it related to a specific group or all groups? |
| Can you tell us more about your experience of using the suctioning catheters once? Did it have an impact on your patient? | Was it effective or resource wasting? |  |
| Can you tell us more about your experience of using the chlorhexidine to flush the suctioning circuit? Did it have an impact on your patient? | Was it effective or resource wasting? |  |

.

**Tool ΙIΙ, Interview Guide, [Part II]: Next of Kin Semi-Structured Interview Questions Tool**

| **Questions** | **Prompts** | **2^nd^ Prompts** |
| --- | --- | --- |
| Think back to your relative and asking you to let him participate in our study. How was your feeling? | Were you motivated to allow his participation?  If no, why? |  |
| In terms of the approach clinical lead nurse/PI used to offer you the deferred consent. Did you feel forced for your choice? | Did you have to sign the consent against your desire? |  |
| Do you have any kind of regression that you allowed your patients participation in the proposed study? | If yes, can you give a reason?  What did you feel? | What did you feel? |
| Were the information sheet that you provided clear for you? | Did you understand the medical terminology used on it? | Did you find a complicated part? |
| Were the health benefits, risks, compensations clear for you? | Did you understand what should be done in case of side effects happened to your relative? |  |
| Did you feel that the proposed intervention affected your relative’s health outcomes? | If yes, Was it in a good or bad way? |  |
