# Supplementary material for: Single‐use versus multiple‐use endotracheal suction catheters flushed with chlorhexidine in mechanically ventilated ICU patients: A study protocol of a feasibility randomized controlled trial with an embedded qualitative study
Source: Nurs Crit Care. 2025 Jan 2;30(4):e13227. doi: 10.1111/nicc.13227 (PMC12234884; doi:10.1111/nicc.13227)
Supplement: Supplementary file 4 — Data S4: Supporting Information. [file NICC-30-0-s003.docx]

# DATA MANAGEMENT PLAN

To ensure General Data Protection Regulations (GDPR) compliance, the University of Plymouth template and guidance for data management plan will be followed including plans for how research data will be collected, managed, stored, used, contextualized, and preserved. It will also provide clarity around issues, such as ownership of data, recovery after a data loss, and data retention policy.

**Data Collection**

We are going to conduct a fRCT with an embedded qualitative design. Therefore, we have two Parts 1) Quantitative and 2) Qualitative.

Part 1

The quantitative study includes mechanically ventilated patients’ data which will be collected within the study setting using data collection tools which have been developed by the PI. Two tools will be used to collect data for this part. The data collected will include sociodemographic characteristics, health relevant data, mechanical ventilator, and endotracheal suctioning data. The CPIS score will be used to evaluate ventilator pneumonia incidence and assessed on first day of ICU Admission then day three and day six. Mechanically ventilated patients will be also evaluated for any other implications of intervention including length of ICU stay, morbidity and mortality.

The data collection tool will be printed and fulfilled for each participant then scanned and uploaded to the University of Plymouth OneDrive.

Part 2

Interviews will be conducted with critical care nurses using a semi-structured interview guide. The interview will be audio- recorded and transcribed verbatim. On transcripts, only the unique identifiers will be used.

**Documentation and Metadata**

The standard file format will be used including audio files from the interviews will be stored in MP3 or WAV format, “Microsoft Word 365” will be used for text-based documents, and “.sav” will be used for SPSS files. Intervention materials including pictures will be stored in PNG. or JPEG format and video files will be in MP4 format.

Anonymised participant data and the semi-structured interviews data will be stored for 10 years in the University of Plymouth OneDrive. These data will be used for data analysis in the final report.

**Ethnics and Legal compliance Informed consent**

Ethical approval will be obtained from the Research Ethical Committee, University of Plymouth, and Mansoura University. An official permission from the hospital's administrative authority to conduct the study will be obtained after explaining the aim and nature of the study. Written informed consent will also be obtained from patients' families (next of kin) by the clinical lead nurse after providing them with complete information regarding the aim of the study, nature of suctioning procedure, expected benefits, and risks. Patients' families will be informed that allowing their patients’ participation in our study is voluntary and refusing to participate will not affect his/her care or treatment. The right to withdraw from the study at any time will be assured without responsibility. Furthermore, they will be informed that confidentiality of patients' personal information will be maintained.

Once they agree participation, three informed consent forms will be provided for signing, dating, and initialling the paper consent forms which will be witnessed by the researcher. One signed copy will be kept in the patient's case notes. Another signed copy will be retained with the PI and last copy for participants’ relative and a photocopy will be retained Mansoura University Emergency Hospital Archive.

**Anonymization**

A unique study identification code will be used to preserve anonymity for patients P01-001 and nurses’ participants N01-001. Relatives’ codes will be R01-001.

**Copyright and** **Intellectual Property Rights (IPR) issues**

Mohamed H. Eid will be the custodian of the data and the University of Plymouth will be the data controller. There is no license or restrictions other than when the research is published. Patenting is not foreseen with this research. Additionally, the researcher (Mohamed H. Eid) is receiving a PhD scholarship from the Ministry of Higher Education, Egypt and the funder will not have intellectual property rights to the data and research findings. In this case, the funder will be acknowledged in any relevant publications.

**Data back up at the University of Plymouth**

The study data will be backed up regularly using Microsoft OneDrive platforms and recovery of the user profile being possible through a restore feature. In the event of a disaster, the University of Plymouth would be consulted to contact Microsoft with support.

**Data access and security**

Access to drives and folders will be password protected and access will be limited to the PI and the research team when necessary and other individuals (such as auditors) authorized by the PI.

**Selection and preservation**

All temporary data such as interview transcripts, and field notes will be transferred to OneDrive at the University of Plymouth for storage in under one week by the custodian Mohamed H. Eid.

**Long-term preservation plan for the dataset**

Completed data collection tools, consent forms, and all data on papers will be scanned and securely stored in a separate folder on the University of Plymouth OneDrive which can only be accessed by the PhD supervisors at the completion of PhD studies. Following transcription and checking the accuracy of the individual interviews, the digital recordings will be deleted. Interview transcripts and field notes will be stored on OneDrive at the University of Plymouth until completion of studies and publication. It would then be retained at the University of Plymouth for 10 years.

**Data Sharing**

The anonymized completed questionnaires, interview transcripts and filed notes will be shared initially with Mohamed H Eid’s PhD supervisory team, using OneDrive for the purposes of this study. Once stored a formal request to share the data will be formally made to the University of Plymouth and permission sought from Mohamed H. Eid or supervisory team. Long term sharing will be managed by the University of Plymouth.

**Restriction on data sharing**

The data will be kept exclusive until the scientific papers are accepted, including the publication of the PhD thesis in the open-access repository of the University of Plymouth.

**Responsibilities and Resources**

Mohamed H. Eid will be the custodian of the generated data, including the implementation of the DMP, data capture, quality, storage, and initial archive. Mansoura University Emergency Hospital will be responsible for the storage of a photocopy of consent forms and patients’ data. The University of Plymouth will maintain the storage of data archive on OneDrive and future sharing.
